# Speckle Vibrometry for Instantaneous Heart Rate Monitoring

**DOI:** 10.3390/s23146312

**Published:** 2023-07-11

**Authors:** Shuhao Que, Fokke van Meulen, Willem Verkruijsse, Mark van Gastel, Sebastiaan Overeem, Sveta Zinger, Sander Stuijk

**Affiliations:** 1 Department of Electrical Engineering, Eindhoven University of Technology, 5600MB Eindhoven, The Netherlands; 2 Kempenhaeghe, 5590AB Heeze, The Netherlands; 3 Philips, 5656AE Eindhoven, The Netherlands

**Keywords:** instantaneous heart rate, inter-beat interval, laser speckle, speckle vibrometry, sleep, contactless, remote

## Abstract

Instantaneous heart rate (IHR) has been investigated for sleep applications, such as sleep apnea detection and sleep staging. To ensure the comfort of the patient during sleep, it is desirable for IHR to be measured in a contact-free fashion. In this work, we use speckle vibrometry (SV) to perform on-skin and on-textile IHR monitoring in a sleep setting. Minute motions on the laser-illuminated surface can be captured by a defocused camera, enabling the detection of cardiac motions even on textiles. We investigate supine, lateral, and prone sleeping positions. Based on Bland–Altman analysis between SV cardiac measurements and electrocardiogram (ECG), with respect to each position, we achieve the best limits of agreement with ECG values of [−8.65, 7.79] bpm, [−9.79, 9.25] bpm, and [−10.81, 10.23] bpm, respectively. The results indicate the potential of using speckle vibrometry as a contact-free monitoring method for instantaneous heart rate in a setting where the participant is allowed to rest in a spontaneous position while covered by textile layers.

## 1. Introduction

Heart rate monitoring plays a vital role in assessing cardiovascular health and understanding physiological responses during sleep. Traditional average heart rate monitoring calculates the mean heart rate over a specific period, providing a general overview of heart activity. However, instantaneous heart rate (IHR) monitoring offers a more detailed and timely assessment by capturing heart rate fluctuations at each moment. Therefore, IHR has more added values than the average heart rate. For example, several decades ago, IHR was already investigated for cyclic sleep phenomena detection [1]. In addition, IHR has also been used for sleep staging [2] and sleep apnea detection [3]. It is thus of significant clinical relevance to measuring IHR during sleep.

Classically, IHR is derived from electrocardiogram (ECG) signals by measuring the time interval between consecutive R peaks [4]. Alternatively, Jose et al. [5] used a copper board with two piezoelectric sensors mounted inside to estimate IHR from a ballistocardiogram (BCG) and subsequently performed heart rate variability analysis. Hernandez et al. [6] used the camera of a smartphone to measure contact-based photoplethysmogram (PPG) and derived IHR, achieving an absolute mean error of 0.94 ± 0.28 beats per minute (bpm). D’Mello et al. [7] used a combination of gyrocardiogram (GCG) and seismocardiogram (SCG) to estimate IHR. Compared to ECG-derived IHR, they achieved 95% limits of agreement of [−8.33, 8.39] bpm with SCG and [−9.29, 10.29] bpm with GCG. Both methods require skin contact, which can cause discomfort while the patient is sleeping. Therefore, for heart rate monitoring during sleep, it is desirable to measure IHR in a contact-free fashion to minimize the interference on the patient.

The existing contact-free sensing modalities for IHR detection include laser Doppler vibrometry (LDV), radiofrequency, and remote PPG. Kroschel et al. [8] used a filtering technique to extract heart sounds from laser Doppler measurements. They only reported performances on the average heart rate extraction. However, since they successfully extracted heart sound peaks, there is potential for LDV to extract IHR. Sakamoto et al. [9] used a millimeter-wave ultra-wideband array radar system to monitor IHR from the human head. Using ECG as the reference, they achieved an average root-mean-square inter-beat interval error of around 16.3 ms from 10 human participants. Iozzia et al. [10] used remote PPG (i.e., camera PPG) to record three regions of interest (ROI) from each subject’s face, including forehead, nose, and cheek. Then, the best ROI was selected to extract IHR from 26 subjects and achieved a mean (standard deviation) error of 0.04 ± 3.29 bpm in a supine position and of 0.01 ± 4.26 bpm in a standing position. Regardless of their contact-free nature, these technologies also have their downsides to consider. LDV is expensive, can only detect motions along the incident laser beam [11], and requires the measured surface to be sufficiently reflective [12]. Remote PPG requires skin exposure, which usually has limited availability in a sleep setting [2]. Radiofrequency technologies in general have difficulty isolating cardiac motions from other detected motions, which renders inferior beat-to-beat measurements [13].

Although the aforementioned existing works have demonstrated the feasibility of extracting IHR in both contact-based and contact-free manners, IHR monitoring remains challenging with existing sensing modalities alternative to ECG because accurate extraction of IHR requires sufficient temporal resolution of the measurement waveform, which remains a non-trivial task.

In this work, we propose a method to measure IHR using speckle vibrometry (SV). SV is a contact-free technology that measures cardiac vibrations remotely by exploiting the laser speckle phenomenon, where a granular pattern will be formed and observed in a defocused camera when a laser beam is illuminated on a surface that is optically rough on the scale of the laser wavelength [14]. This pattern is very sensitive to minute motions and thus will change according to each vibration [15].

SV has already been investigated to extract average heart rate by Havakuk et al. [16] and by our previous work [17]. Havakuk et al. collected a 3 min continuous recording from the left side of the chest of each subject in a sitting position. From the recording, they extracted average heart rate from 1 min windows and achieved an error of around 1 bpm compared with ECG. Their measurements excluded the use of reflective and highly textured textiles and their results focused on on-skin measurements. By contrast, our previous work demonstrated that SV could extract average heart rate from several spots in the thoracic area (i.e., in a supine position) even when thick layers of textile (up to 32 mm) were on top of the body, including smooth cotton and highly textured furry materials. However, each unique continuous measurement was only 1 min long, which was not sufficient for the investigation of IHR performance. Moreover, only the supine position with measurement spots adjacent to the heart was investigated. Apart from measurement spots that are in the thoracic area, SV has also been used by Beiderman et al. [18] to extract BCG from the wrist. The peak amplitudes of the measured BCG were then used to estimate blood pressure values. Although existing works have demonstrated the feasibility of using SV for the extraction of average heart rate from the chest and even of BCG from the wrist, research into IHR extraction and other relevant measurement spots is still missing, which is important for monitoring heart rate during sleep where the subject’s thoracic area and wrist are not always available overnight.

In this work, fixing the positioning of the camera and the laser, we investigate three sleeping positions: supine, lateral, and prone. The measurement spots are, respectively, on the sternum, left arm, and left scapular, with the latter two being distant from the heart. Each unique measurement has a duration of 5 min, which is sufficient for IHR extraction and evaluation. In order to complete an inter-beat interval comparison between ECG and SV measurements, we propose a method to match the R peaks of ECG with their corresponding aortic valve opening (AO) peaks of SV, which also allows us to evaluate our beat detection performance.

## 2. Materials and Methods

In this section, we introduce the working principle of SV and the deployed devices, the dataset that we used, the beat detection and selection algorithms that we implemented for SV and ECG, and the evaluation metrics that we employed.

### 2.1. SV & Device Specifications

**Figure 1:**
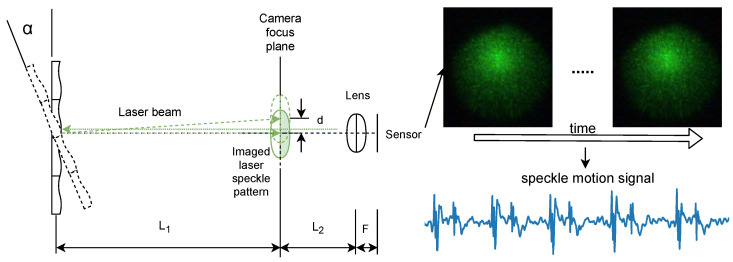
Schematic of a near-focused camera system for speckle vibrometry [17].

Following the same design in our previous work [17], our SV setup comprises a near-focused camera and a laser source, as shown in Figure 1. $L_{1}$ denotes the distance between the measured surface to the focal plane of the camera. $L_{2}$ denotes the distance between the camera focal plane and the camera lens. The camera is said to be near-focused when the camera focal plane is located between the measured surface and the camera lens [19]. Under the said imaging condition, neither transversal nor axial movement affects the distribution of the speckle pattern [15]. As a result, the tilt of the measured surface is magnified significantly [19] and manifests as displacements of the imaged speckle pattern, according to the following equation:

(1) $$d=\frac{L_{1}F}{L_{2}}\tan\alpha$$

where *d* denotes a relative shift in the speckle pattern due to surface tilt, α denotes the tilt angle of the surface, and *F* denotes the focal length of the imaging lens.

To extract the displacement between laser speckle patterns of consecutive video frames, we employed the sub-pixel image registration algorithm proposed by Guizar-Sicairos et al. [20] with an up-sampling factor equal to 100 (i.e., a sub-pixel accuracy of 0.01 pixels) that was empirically selected to guarantee the detection of minute cardiac motions. In our setup, we have the values of $L_{1}$, $L_{2}$, and *F*, respectively, equal to 0.8 m, 0.4 m, and 0.05 m, which renders a minimal detectable angular change of 0.0001 pixels/mm, corresponding to a displacement of 0.01 pixels in an SV measurement. By motion extraction, displacements between consecutive video frames along two directions, the *x*-axis and *y*-axis, are extracted. Based on the method proposed by our previous work [17], we derived a single measurement for SV denoted as $D_{r}$, which is independent of the camera surface orientation.

**Figure 2:**
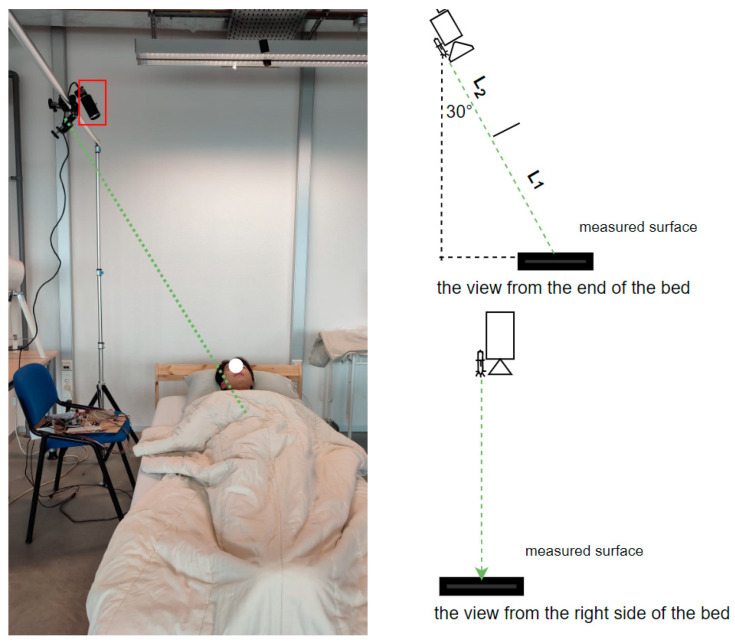
The illustration of the experimental setup of speckle vibrometry. The two figures on the right are illustrations of the camera and laser positioning from two view angles: from the end of the bed and from the right side of the bed. The elevation angle of the camera and laser is around 120 degrees. The figure on the left is the real-life experimental setup that we deployed for data acquisition, where the green dashed line indicates the laser beam and the red rectangle indicates the camera. This configuration is applied to all three sleeping positions: supine, lateral, and prone.

The experimental setup is illustrated in Figure 2. A monochrome camera with a 2.35 megapixel CMOS sensor (UI-3060CP-M-GL, IDS Imaging Development Systems GmbH, Obersulm, Germany) was used. During measurement, the camera operated at a frame rate of 300 frames per second with an exposure time of 1.000 ms. An Aixiz Class-I green laser (AD-532-1-830) was mounted in parallel with a C-mount camera lens (M111FM50, Tamron, Saitama, Japan). The laser light source has a wavelength of 532 nm and an emission power of <1 mW. The camera lens’ aperture (f-stop) was set at F/1.8. The distance between the camera and the bed surface was 1.5 m, rendering the measuring distance $L_{1}+L_{2}$ approximately 1.2 m. For the single-lead ECG signal acquisition, we used a DFRobot Heart Rate Monitor Sensor (SEN0213) connected to an Arduino Uno board, which was operated at a sampling frequency of 300 Hz. Data from different devices were synchronized with timestamps.

### 2.2. Dataset

After the study was approved (ERB2022EE1) by the Ethical Review Board of Eindhoven University of Technology, the Netherlands, we collected data from 20 participants at the sleeping lab. Among the 20 participants, there are 8 females and 12 males. The age range of female participants is between 18 and 28, while that of male participants is between 18 and 39. The BMI range of female participants is between 17.4 and 24.5, while that of male participants is between 17.8 and 37.3. For each participant, we measured while the participant was in three different sleeping positions (supine, lateral, and prone). While in these positions, we measured the sternum, left arm, and left scapular, respectively. For each sleeping position, we varied the use of 5 different textile layers: no textile layer, bedsheet layers of 8 mm, 16 mm, 32 mm thick, and a comforter layer of 32 mm thick. This resulted in 15 unique measurements per participant. Each measurement includes 1 video recording for SV and 1 single-lead ECG recording. During the recording, the participant was instructed to breathe spontaneously. The used bedsheets are composed of cotton material and white color. The ticking of the used comforter is woven cotton and of white color and the filling power is synthetic fibers.

### 2.3. Beat Detection and Selection and Inter-Beat Interval Selection

To extract cardiac peaks in $D_{r}$, we employed the peak detection algorithm proposed by our previous work [17] with some modifications. In [17], we set the peak amplitude threshold to 0 when detecting peaks in the principal signal, which is defined as the band-pass filtered (0.5–3 Hz) Hilbert envelope of the original SV signal. In this work, inspired by Pan et al. [21], we employed adaptive thresholding with 5 s non-overlapping windows. This addition made the algorithm more robust against detecting false peaks in the principle signal. The details are presented in Appendix A.

To extract R peaks in ECG, we implemented a widely adopted method proposed by W. Engelse and C. Zeelenberg [22] with added modifications proposed by A. Lourenco et al. [23].

In order to compare the instantaneous heart rates obtained from ECG and SV, it is necessary to ensure that the beat-to-beat intervals measured from both methods correspond exactly. Prior to that, we first located the one-to-one mapping between ECG R peaks and SV cardiac peaks. We extracted valid pairs of cardiac peaks from both ECG and SV waveforms to achieve this. For each detected ECG R peak, within a given interval, we locate the corresponding detected SV cardiac peak. If there is one SV cardiac peak detected within that interval, we select it. If there is more than one SV cardiac peak detected within that interval, we select the one that is the most adjacent to the R peak and discard the rest (i.e., false positive peaks). If there is no SV cardiac peak detected, we discard the R peak (i.e., a false negative peak). Finally, we append the R peak and the corresponding SV cardiac peak to $M_{valid}$ and $N_{valid}$, respectively. The details of determining the interval are presented in Algorithm 1. It is worth mentioning that, instead of using 0.5, which indicates half the distance between the current peak and the previous peak, and that between the current peak and the next peak, we used 0.4 to impose a more stringent and narrower interval.

To quantify the SV-based cardiac beat detection performance, we define two evaluation metrics, precision and recall, as follows:

(2) $$recall=\frac{\left|M_{valid}\right|}{\left|N_{ECG}\right|}$$

(3) $$precision=\frac{\left|N_{valid}\right|}{\left|N_{SV}\right|}$$

where $\left|N_{ECG}\right|$ denotes the number of R peaks detected in ECG, $\left|N_{SV}\right|$ denotes the number of cardiac peaks detected in an SV measurement, $\left|N_{valid}\right|$ denotes the number of cardiac peaks in $N_{valid}$, and $\left|M_{valid}\right|$ denotes the number of R peaks in $M_{valid}$.

After obtaining one-to-one mapping between ECG R peaks ($M_{valid}$) and SV cardiac peaks ($N_{valid}$), we derived the inter-beat intervals from both measurements by calculating the differences between consecutive peaks, respectively, from $M_{valid}$ and $N_{valid}$. Based on the ground truth peaks detected from ECG ($N_{ECG}$), we labeled as “false intervals” the inter-beat intervals calculated from $M_{valid}$ where there existed one R peak in the original location that was discarded during beat selection, and we discarded the corresponding inter-beat intervals calculated from $N_{valid}$. This inter-beat interval selection avoids overestimating the performance of SV by excluding the “false intervals” that were incurred by missed detected SV cardiac peaks (i.e., false negative peaks). In this way, we derived one-to-one mapping of inter-beat intervals between ECG and SV measurements, denoted as $IBI_{ECG}$ and $IBI_{SV}$, respectively.
**Algorithm 1** Derivation of valid pairs of cardiac peaks from ECG($M_{valid}$) and SV($N_{valid}$)$\left|N_{ECG}\right|$                    ▹ number of detected peaks in ECG$\left|N_{SV}\right|$                      ▹ number of detected peaks in SV$N_{SV}\leftarrow \left[n_{1},\ldots ,n_{i},\ldots ,n_{\left|N_{SV}\right|}\right]$             ▹ detected cardiac peaks in SV$M_{ECG}\leftarrow \left[m_{1},\ldots ,m_{j},\ldots ,m_{\left|N_{ECG}\right|}\right]$            ▹ detected cardiac peaks in ECG$N_{valid}\leftarrow \left[\right]$                ▹ the initial list of valid cardiac peaks in SV$M_{valid}\leftarrow \left[\right]$               ▹ the initial list of valid cardiac peaks in ECG**while** $j\leq \left|N_{ECG}\right|$ **do**    **if** j=1 **then**        $Interval\leftarrow [0,m_{j}+0.4*\left(m_{j+1}-m_{j}\right)]$    **else if** $j<\left|N_{ECG}\right|$ **then**        $Interval\leftarrow [m_{j}-0.4*\left(m_{j}-m_{j-1}\right),m_{j}+0.4*\left(m_{j+1}-m_{j}\right)]$    **else**        $Interval\leftarrow [m_{j}-0.4*\left(m_{j}-m_{j-1}\right),m_{j}]$    **end if**    **if** there is at least one $n_{i}$ in Interval **then**        $n_{i}\leftarrow \arg\min_{i}\left(\right|m_{j}-N_{SV}\left|\right)$        **if** $n_{i}$ not in $N_{valid}$ **then**           $N_{valid}\leftarrow n_{i}$                 ▹$n_{i}$ is appended to the list $N_{valid}$           $M_{valid}\leftarrow m_{j}$                ▹$m_{j}$ is appended to the list $M_{valid}$        **end if**    **end if**    j←j+1**end while**

### 2.4. Evaluation Metrics

To evaluate the performance of SV on IHR extraction, we employed the Bland–Altman analysis [24] to quantify the agreement between SV cardiac measurements and ECG, based on mean bias ± standard deviation (SD) and limits of agreement (LOA). To investigate the impact of gender and body mass index (BMI) on the IHR performance of SV, we compared the average absolute instantaneous heart rate difference between ECG and SV. For the statistical significance analysis of gender, we employed the Kruskal–Wallis H-test [25] because it does not assume a normal distribution. For BMI, due to an imbalanced distribution between BMI > 25 (5 participants) and BMI ≤ 25 (15 participants), we employed the Welch’s *t*-test [26], commonly used for unequal sample sizes. In order to verify the validity of Welch’s *t*-test and Bland–Altman analysis, the normality of the datasets’ distributions was assessed by the Shapiro–Wilk test [27] with *p*-value equal to 0.05 as the significance level.

## 3. Results

In this section, we show the SV cardiac waveform in the time domain, present the beat detection results, and evaluate the IHR extraction performance of SV under different recording conditions.

### 3.1. SV Cardiac Waveform in the Time Domain

**Figure 3:**
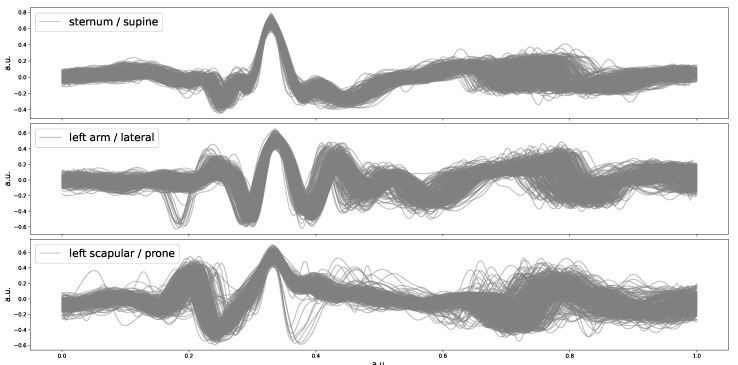
Ensemble average of SV cardiac waveforms from one participant in the time domain. The *x*-axis and *y*-axis are both normalized, rendering an arbitrary unit (a.u.).

After peak detection, we performed ensemble averages over three 5 min SV measurements for each participant that correspond, respectively, to supine, lateral, and prone positions, with the purpose of showing the SV cardiac waveform in the time domain. The ensemble result for one of these participants is presented in Figure 3. As can be observed, for this participant, when the sleeping position is supine, the SV waveform variations appear to be the least and exhibit the strongest peak prominence.

### 3.2. Beat Detection Performance

Table 1 presents the detection performance of the cardiac beats extracted from SV with respect to three sleeping positions. It can be observed that the best beat detection performance was when the sleeping position was supine. The differences between the three sleeping positions are no more than 3% in terms of mean precision and recall values, indicating that the SV signal qualities do not differ too much in different positions, which is consistent with the example SV cardiac waveforms presented in Figure 3.

### 3.3. Instantaneous Heart Rate Extraction

To compare instantaneous heart rates extracted, respectively, from ECG and SV, we used $IBI_{ECG}$ and $IBI_{SV}$ after one-to-one mappings of the inter-beat intervals between ECG and SV (see Algorithm 1).

**Figure 4:**
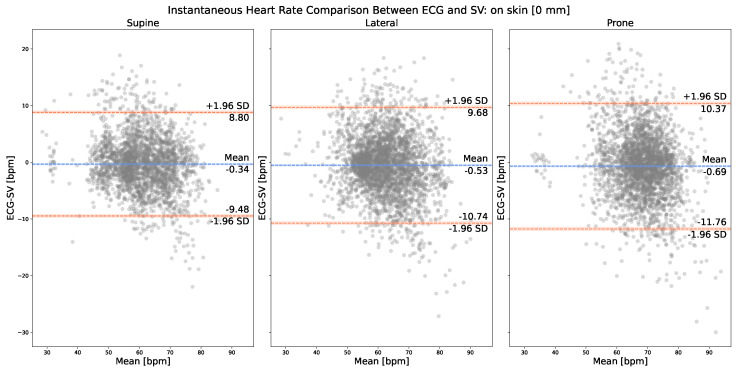
Bland–Altman plot: on-skin IHR comparison between SV (i.e., $N_{valid}$) and ECG (i.e., $M_{valid}$).

Figure 4 presents the on-skin IHR comparison between the corresponding cardiac peaks, respectively, from $M_{valid}$ and $N_{valid}$. The normality of the IHR differences was verified by the Shapiro–Wilk test, with the resulting p-values more than 0.05 for all three plots. As can be observed, in the case of on-skin SV measurements, the best LOA [−9.48, 8.80] bpm was achieved with the supine position (i.e., sternum). In terms of mean bias, the supine position also yielded the smallest bias, −0.34 bpm. Our SV measurement system appears to slightly overestimate IHR values (<1 bpm) compared with ECG, as indicated by the mean negative bias values obtained with all three anatomical locations, −0.34 bpm, −0.53 bpm, and −0.69 bpm.

**Figure 5:**
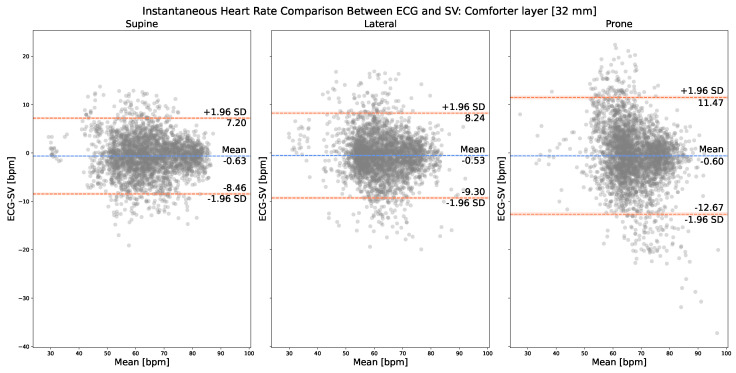
Bland–Altman plot: on-comforter (32 mm) IHR comparison between SV (i.e., $N_{valid}$) and ECG (i.e., $M_{valid}$).

Figure 5 presents the on-comforter IHR comparison between the corresponding cardiac peaks, respectively, from $M_{valid}$ and $N_{valid}$. The normality of the IHR differences was verified by the Shapiro–Wilk test, with the resulting p-values more than 0.05 for all three plots. As can be observed, in the case of on-comforter SV measurements (i.e., with 32 mm of layer thickness), the best LOA [−8.46, 7.20] bpm was achieved with the supine resting position, which is comparable to and does not yield a large difference (around 1 bpm) from its corresponding on-skin measurements.

**Figure 6:**
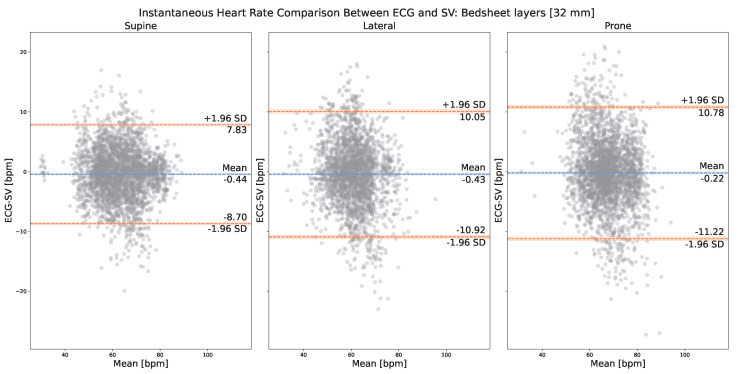
Bland–Altman plot: on-bedsheets (32 mm) IHR comparison between SV (i.e., $N_{valid}$) and ECG (i.e., $M_{valid}$).

Figure 6 presents the on-bedsheets IHR comparison between the corresponding cardiac peaks, respectively, from $M_{valid}$ and $N_{valid}$. The normality of the IHR differences was verified by the Shapiro–Wilk test, with the resulting p-values more than 0.05 for all three plots. As can be observed, in the case of on-comforter SV measurements (i.e., with 32 mm of layer thickness), the best LOA [−8.70, 7.83] bpm was achieved with the supine resting position, which is comparable to and does not yield a large difference (around 1 bpm) from its corresponding on-skin measurements.

To evaluate if adding textile layers of a certain thickness can yield better results than on-skin and on 32 mm layer measurements, we generated additional results for 8 mm bedsheet layers and 16 mm bedsheet layers, as presented in Table 2. By looking at Table 2 and Figure 4 and Figure 6, it can be observed that, for both the supine and prone positions, the best IHR performance was achieved when there were 8 mm thick bedsheet layers. For the lateral position, the best IHR performance was achieved when there were 16 mm thick bedsheet layers. However, the margins of improvement are all very small, less than 1 bpm in terms of both mean bias ± SD and LOA.

Regardless of the presence of textile layers, the supine position yielded the best performance in terms of LOA, lateral the second, and prone the last. For each sleeping position, supine, lateral, and prone, we only found small differences (<2 bpm) among on-skin, on-comforter (32 mm), and on-bedsheets (32 mm) SV measurements in terms of their mean bias ± SD and LOA with ECG. This result indicates that SV measurement is not significantly impacted by the presence of 32 mm thick textile layers compared with no layer, regardless of their materials, with the comforter being more fluffy and airy and the bedsheet being more rigid.

We found no statistically significant differences between female and male participants regardless of the measured anatomical locations or applied textile layers. In terms of the impact of a participant’s BMI, after using the Shapiro–Wilk test to verify the normality of the distributions of ECG-SV IHR differences, we also found no statistically significant differences.

## 4. Discussion

SV is a feasible method for the estimation of IHR of participants in different sleeping positions, and the results are not significantly influenced by the use of different layers of bedsheets or one layer of a comforter.

Our experiment principally investigated two important variables that are found in a sleep scenario: sleeping positions and the use of textile layers. On-skin measurement results showed that SV could measure IHR in all three common sleeping positions, supine, lateral, and prone, with the measured anatomical locations being the sternum, left arm, and left scapular, respectively. This indicated that SV is not too sensitive (around 1 bpm of difference) to the measured location of the human body in terms of detecting IHR even when the location is distant from the heart (i.e., left arm and left scapular), which is a useful feature for monitoring heart rate during sleep where patients can have spontaneous movements and sleep in their desired posture overnight. However, since SV is a motion sensor, it is sensitive to motion distortions. If the patient only moves occasionally during sleep, then we can use a motion distortion detection algorithm to remove those episodes. If the patient moves constantly during sleep, which is highly unlikely, then a new algorithm that can extract IHR from contaminated SV measurements needs to be proposed. On-textile measurement results showed that SV could measure IHR on two commonly used textile materials: bedsheets and comforters, with the former being more rigid and the latter being more airy and fluffy. Its performance withstands even when the textile layer thickness reaches 32 mm, which is relevant and useful for monitoring heart rate during sleep when patients use blankets in winter. However, it is worth noting that, in terms of LOA, the supine position yielded the best performance in all the aforementioned cases. We expect that it is due to the fact that, in the supine position, the measured anatomical location, the sternum, is the closest to the heart compared with the left arm (lateral) and the left scapular (prone). The performance differences between supine and lateral/prone are around 1 bpm for on-skin measurements, around 1 bpm/4 bpm for on-comforter measurements, and around 2 bpm for on-bedsheets measurements. These differences are not large except between supine and prone when there is one layer of comforter (32 mm thick), in which case SV still achieved a mean bias ± SD value of −0.60 ± 6.04 bpm with the prone position. We speculate that, in the prone position, compared with the supine position, it is harder for cardiac vibrations to propagate through the comforter layer.

We hypothesized that adding a certain number of textiles could improve the SV’s performance even better than on-skin measurements. We experimented with 8 mm and 16 mm thick bedsheet layers. The results indicated a slight improvement (less than 1 bpm) in terms of SD and LOA for supine and prone positions with 8 mm thick bedsheet layers, and for the lateral position with 16 mm thick bedsheet layers. We speculated that bedsheet layers could dampen large motions manifested on the skin surface that are incurred by respiration, subsequently allowing for the cardiac vibrations to be extracted more easily. Further investigations are needed to locate a more precise layer thickness value that could be potentially optimal for SV to extract IHR and to evaluate the impact of other textile materials.

Our participant group includes both male and female participants, with varying BMI values. Our investigations found no significant impact of either gender or BMI on SV’s performance. This result showed that SV could potentially be used on a wide range of patients.

Our current SV setup employs a green laser light source with an emission power of less than 1 mW, which is considered class-I and safe. No other visible light source is needed in the room. Such dim laser light should not cause any discomfort to patients in a dark room. Nevertheless, in the future, we plan to investigate the usage of invisible light, such as near-infrared light. Whether the performance will be compromised or not by using a near-infrared laser source still awaits further investigations.

However, there are other important variables in monitoring IHR during sleep that we have not investigated in this work, including auto-aiming of the camera and the laser, overnight recording (e.g., a continuous recording of 24 h), impact of abnormal breathing patterns, and impact of abnormal heart rates. Although our results already showed that SV is not sensitive to the measured anatomical location and can measure heartbeats even on textile layers, rendering a more relaxed aiming requirement, it is still imperative to investigate if SV can measure heartbeats when the laser spot is aimed at anatomical locations that are very distant from the heart, such as the foot. It will also be interesting to investigate if SV can measure heartbeats on parts of textiles that have no direct contact with the human body. Since our participants for this study are healthy volunteers, there is a lack of variety in terms of breathing patterns and heart rates. For future work, we should focus on real patients and investigate if the performance of SV on IHR is influenced by abnormal breathing patterns or abnormally high heart rates. Other than monitoring heart rate during sleep, SV as a contact-free sensing modality also has other potential applications, such as monitoring patients in a post-operation room or patients with burned skin that can react negatively to ECG electrodes. However, these application scenarios also pose new challenges (e.g., different positions, and more frequent patient movement) that we need to investigate in the future.

## 5. Conclusions

IHR is an important physiological parameter to monitor for sleep medicine. In a sleep setting, a contact-free sensing modality is more desirable than contact-based ones because it introduces less disturbance to and constraint on patients’ spontaneous movement. As a contact-free technology, SV proves to be able to measure IHR on multiple anatomical locations of the human body, including the sternum, left arm, and left scapular. Its performance does not deteriorate with the application of textile layers up to a thickness value of 32 mm. Neither the BMI nor gender exhibit a significant impact on said ability. Our results indicate the potential of using SV as a contact-free modality to monitor IHR in a sleep setting. Further research is needed to discover the optimal laser spot positioning for SV. Our future work will concentrate on its clinical applications.

## Figures and Tables

**Table 1:** SV-based cardiac beat detection performance. The results are presented in the form of mean and standard deviation (SD) values of 300 unique 5 min measurements (i.e., 15 unique measurements per participant).

Mean (SD)	Supine	Lateral	Prone
Recall (%)	84 (10)	81 (9)	81 (12)
Precision (%)	83 (9)	81 (8)	80 (10)

**Table 2:** IHR performance on three resting positions with different bedsheet layer thicknesses, represented by mean bias ± standard deviation (SD) and limits of agreement (LOA) derived from Bland-Altman analysis.

Mean Bias ± SD LOA [bpm]	8 mm	16 mm
Supine	−0.43 ± 4.19	−0.45 ± 4.54
	−8.65, 7.79	−9.35, 8.45
Lateral	−0.47 ± 5.08	−0.27 ± 4.86
	−10.44, 9.49	−9.79, 9.25
Prone	−0.29 ± 5.37	−0.17 ± 5.63
	−10.81, 10.23	−11.21, 10.87

## Data Availability

Data sharing is not applicable to this article.

## Abbreviations

The following abbreviations are used in this manuscript:

IHR	Instantaenous heart rate
BPM	Beats per minute
SV	Speckle vibrometry
BMI	Body mass index
SD	Standard deviation
LOA	Limits of agreement
ECG	Electrocardiogram
BCG	Ballistocardiogram
PPG	Photoplethysmogram
SCG	Seismocardiogram
GCG	Gyrocardiogram
LDV	Laser Doppler vibrometry

## Appendix A

**Algorithm A1** Adapted thresholds for the principle signal of SV
$\left|S_{SV}\right|$ ▹ number of non-overlapping 5 s windows $S_{SV}\leftarrow \left[s_{1},\ldots ,s_{i},\ldots ,s_{\left|S_{SV}\right|}\right]$ ▹ segments of the principle signal of SV $find\_peaks(A_{1},A_{2})$ ▹ peak detection algorithm, where $A_{1}$ denotes the input signal and $A_{2}$ denotes the threshold **while** $i\leq \left|S_{SV}\right|$ **do** **if** i=1 **then** $Bins\leftarrow [b_{1},b_{2},\ldots ,b_{100}]$ ▹$b_{1}=min\left(s_{i}\right)$, $b_{100}=max\left(s_{i}\right)$ $Hist\leftarrow Histogram(s_{i})$ ▹ the histogram of $s_{i}$ $Thres\leftarrow \frac{\left(Bins\left[\arg\max_{Hist}\right]+Bins\left[\arg\max_{Hist}-1\right]\right)}{2}+\frac{\sqrt{mean(s_{i}^{2})}}{2}$ ▹ the initial threshold $Beats\leftarrow find\_peaks\left(s_{i},Thres\right)=\left[p_{1},\ldots ,p_{m}\right]$ ▹ the detected m peaks $IBI\leftarrow mean(\left[p_{2}-p_{1},\ldots ,p_{m}-p_{m-1}\right])$ ▹ the average inter-beat intervals $Thres_{prev}\leftarrow Thres$ ▹ update the previous threshold value $IBI_{prev}\leftarrow IBI$ ▹ update the previous average inter-beat interval value **else** $Beats\leftarrow find\_peaks\left(s_{i},Thres_{prev}\right)=\left[p_{1},\ldots ,p_{m}\right]$ **while** m < 2 **do** $Thres_{prev}=Thres_{prev}*0.99$ $Beats\leftarrow find\_peaks\left(s_{i},Thres_{prev}\right)=\left[p_{1},\ldots ,p_{m}\right]$ **end while** $IBI\leftarrow mean(\left[p_{2}-p_{1},\ldots ,p_{m}-p_{m-1}\right])$ **if** $\max IBI>1.5*IBI_{prev}$ **then** β←0.25 **else if** minIBI<0.4 s **then** β←0.125 **else** β←0 **end if** $Thres\leftarrow \beta *mean\left(s_{i}\left[p_{1},p_{2},\ldots ,p_{m}\right]\right)+\left(1-4*\beta \right)*Thres_{prev}$ $Beats\leftarrow find\_peaks\left(s_{i},Thres\right)=\left[p_{1},\ldots ,p_{m}\right]$ $Thres_{prev}\leftarrow Thres$ ▹ update the previous threshold value $IBI_{prev}\leftarrow IBI$ ▹ update the previous average inter-beat interval value **end if** **end while**
